# Plasma lipid metabolites as potential biomarkers for identifying individuals at risk of obesity-induced metabolic complications

**DOI:** 10.1038/s41598-023-38703-8

**Published:** 2023-07-20

**Authors:** Paula Emília Nunes Ribeiro Bellot, Erik Sobrinho Braga, Folorunsho Bright Omage, Francisca Leide da Silva Nunes, Severina Carla Vieira Cunha Lima, Clélia Oliveira Lyra, Dirce Maria Lobo Marchioni, Lucia Fatima Campos Pedrosa, Fernando Barbosa, Ljubica Tasic, Karine Cavalcanti Maurício Sena-Evangelista

**Affiliations:** 1 Postgraduate Program in Nutrition, Center for Health Sciences, Federal University of Rio Grande do Norte, Natal, Rio Grande do Norte Brazil; 2 Biological Chemistry Laboratory, Department of Organic Chemistry, Institute of Chemistry, University of Campinas (UNICAMP), Campinas, São Paulo Brazil; 3 Computational Biology Research Group, Embrapa Agricultural Informatics, Campinas, São Paulo Brazil; 4 Department of Nutrition, Federal University of Rio Grande do Norte, Natal, Rio Grande do Norte Brazil; 5 Department of Nutrition, School of Public Health, University of São Paulo, São Paulo Campus, São Paulo, SP Brazil; 6 Department of Clinical Analyses, Toxicology and Food Sciences, School of Pharmaceutical Sciences of Ribeirão Preto of the University of São Paulo, Ribeirão Preto, São Paulo, Brazil

**Keywords:** Obesity, Lipidomics

## Abstract

Lipidomics studies have indicated an association between obesity and lipid metabolism dysfunction. This study aimed to evaluate and compare cardiometabolic risk factors, and the lipidomic profile in adults and older people. A cross-sectional study was conducted with 72 individuals, divided into two sex and age-matched groups: obese (body mass index—BMI ≥ 30 kg/m^2^; n = 36) and non-obese (BMI < 30 kg/m^2^; n = 36). The lipidomic profiles were evaluated in plasma using ^1^H nuclear magnetic resonance (^1^H-NMR) spectroscopy. Obese individuals had higher waist circumference (p < 0.001), visceral adiposity index (p = 0.029), homeostatic model assessment insulin resistance (HOMA-IR) (p = 0.010), and triacylglycerols (TAG) levels (p = 0.018). ^1^H-NMR analysis identified higher amounts of saturated lipid metabolite fragments, lower levels of unsaturated lipids, and some phosphatidylcholine species in the obese group. Two powerful machine learning (ML) models—*k*-nearest neighbors (*k*NN) and XGBoost (XGB) were employed to characterize the lipidomic profile of obese individuals. The results revealed metabolic alterations associated with obesity in the NMR signals. The models achieved high accuracy of 86% and 81%, respectively. The feature importance analysis identified signal at 1.50–1.60 ppm (–CO–CH_2_–CH_2_–, Cholesterol and fatty acid in TAG, Phospholipids) to have the highest importance in the two models.

## Introduction

Obesity is a complex multifactorial disease associated with an increased risk of several non-communicable diseases. The worldwide prevalence of obesity is approximately 13% of the adult population, contributing to 2.8 million deaths due to excess weight^1^. Evidence has suggested that obesity is associated with adipose tissue dysfunction, such as adipocyte hypertrophy, hypoxia, and a shift towards generating proinflammatory adipose tissue that drives the development of dyslipidemia and insulin resistance. This scenario requires early identification of metabolic changes through biomarkers to prevent obesity-associated complications^2^.

Obesity-induced dyslipidemia involves several pathophysiological mechanisms with distinct characteristics depending on many individual factors^3^. Usually, obese patients have elevated levels of both fasting and postprandial triacylglycerols (TAG), elevated free fatty acids (FA), reduced high-density lipoprotein cholesterol (HDL-C), high-normal to moderately increased low-density lipoprotein cholesterol (LDL-C), and elevated small dense LDL. This subtype of dyslipidemia is generally termed metabolic-related dyslipidemia^4^. Nonetheless, there are some obese patients in whom dyslipidemia is less prominent and even absent, and there are still gaps in the actual risk of this current "metabolically healthy" state in this group^5^.

The routine lipid analyses that are currently used to assess the lipid profile for the diagnosis of dyslipidemia are often criticized as they can only measure total lipid concentrations without identifying individual lipid species that contribute to these concentrations^6^. It is important to identify these species because depending on the length of the carbon chain and the degree of desaturation of FA, these compounds may positively or negatively influence the production of inflammatory markers related to the development of noncommunicable diseases in overweight and obese individuals^7^.

Additionally, the origin of lipids, whether synthesized endogenously or obtained from the diet, influences their accumulation and metabolism, and consequently, their biological functions^8^. Thus, dietary FAs may occur in the free form in the plasma, or as part of complex molecules that represent the lipidomic profile and subsequent health effects^9^. Saturated fatty acids (SFAs) induce stress and activate the inflammatory cascade, exacerbating insulin resistance. For example, polyunsaturated FAs (PUFAs) n-3 act on pathways that induce anti-inflammatory properties and have been associated with improved insulin sensitivity, resting blood pressure, and reduced triglyceridemia, while n-6 PUFA is associated with lower levels of plasma LDL-C, showing inverse relationship with the risk of cardiovascular disease^10–12^. In addition, monounsaturated FAs (MUFAs) have been associated with increased HDL-C levels and improved insulin sensibility^10^.

Lipidomics can provide a range of information, allowing us to understand lipid changes related to the pathogenesis of metabolic diseases, such as obesity. A previous 5-year follow-up study demonstrated the relationship between the baseline lipidomic profile and changes in waist circumference (WC) and body mass index (BMI); for example, alkyl-diacylglycerol displayed the strongest positive association with changes in WC, although some unsaturated FAs were negatively associated with both WC and BMI gain^13^. Another study identified that plasma lipid metabolites, such as some lysophosphatidylcholines and several acylalkylphosphatidylcholines, were also inversely correlated with BMI, demonstrating differences among subjects with different classes of obesity^14^. Furthermore, in a study of obese patients undergoing bariatric surgery, lipidomic analysis conducted using the nuclear magnetic resonance (NMR) technique demonstrated a reduction in a biomarker of cholesterol production rate, in addition to quantitative changes in many groups of polar lipids, including phosphatidylcholine (PC), phosphatidylethanolamine, sphingomyelin, and total phospholipids (PL), as well as unsaturated FAs^6^.

Authors believe that NMR-based techniques are powerful tools to perform lipidomics analyses because of their advantages when compared with other methodologies^15^. For example, the sample is not destroyed or chemically altered after the analyses; it has high analytical reproducibility; easily identifies molecular moieties; it presents high robustness of instruments and offers the possibility to obtain molecular dynamics information and direct quantitative information; also, it doesn’t require specific standards for quantification. Despite the fact that NMR-based methods have lower sensitivity when compared to liquid chromatography – mass spectrometry (tandem) (LC-MS/MS), this last methodology requires laborious work on sample preparation, fractionation, and analysis of different sample fractions separately by applying different hyphenated techniques liquid chromatography (LC) or gas chromatograph (GC) before the proper MS/MS analysis^16^.

Therefore, this study was conducted to evaluate and compare the cardiometabolic risk factors, and lipidomic profile of obese and non-obese adults and older people by using ^1^H-NMR and machine learning (ML) tools. It is an original study, the first to perform lipidomic analysis in the population, and it could help to bring new light to the issue of the early definition of the risk of metabolic complications induced by obesity by identifying new biomarkers.

## Results

### Characteristics of study population

The main characteristics of the study population are summarized in Table 1. The median age was 62 (48.8–68.0) years, and 66.7% of the individuals were women. More than 60% of the population had never smoked or consumed alcohol, and 73.6% had dyslipidemia. Individuals with obesity had a higher frequency of metabolic syndrome (p = 0.004), and significantly higher values of WC (p < 0.001) and visceral adiposity index (VAI) (p = 0.029). The homeostatic model assessment insulin resistance (HOMA-IR) (p = 0.010) and TAG (p = 0.018) levels were higher in the obese group. The global risk score was also different between the groups, with 86.1% of the obese individuals showing a high cardiovascular risk (p = 0.009). However, blood pressure measurements did not differ between the groups.

**Table 1:** Socio-biodemographic, clinical and biochemical characteristics of participants.

Variable	Total (n = 72)	Non-obese (n = 36)	Obese (n = 36)	p-value
Age (years)^b^	62 (48.8–68.0)	62.5 (49.0–67.5)	61 (48.0–68.0)	0.813
Sex^c^				
Male	24 (33.3)	12 (33.3)	12 (33.3)	1.00
Female	48 (66.7)	24 (66.7)	24 (66.7)	
Lifestyle habits^c^				
Never smoked	45 (62.5)	22 (61.1)	23 (63.9)	0.817
Non drinker	44 (61.1)	22 (61.1)	22 (61.1)	0.497
NCD^c^				
Arterial hypertension	33 (45.8)	14 (38.9)	19 (52.8)	0.488
Type 2 diabetes	15 (20.8)	9.0 (25.0)	6.0 (16.7)	0.683
Dyslipidemia	53 (73.6)	24 (66.7)	29 (80.6)	0.181
Metabolic syndrome	40 (55.6)	14 (38.9)	26 (72.2)	**0.004**
Stroke/heart attack	5.0 (6.9)	3.0 (8.3)	2.0 (5.6)	0.643
Global Risk Score^c^				
Low risk	13 (18.1)	10 (27.8)	3.0 (8.3)	
Moderate risk	7.0 (9.7)	5.0 (13.9)	2.0 (5.6)	**0.009**
High risk	49 (68.0)	18 (50.0)	31 (86.1)	
Very high risk	3.0 (4.2)	3.0 (8.3)	0.0 (0.0)	
Waist circumference^a^	98.8 (13.9)	90.5 (11.1)	108.8 (9.8)	**< 0.001**
Visceral adiposity index^b^	2.5 (1.6–4.4)	2.2 (1.4–3.9)	3.1 (1.7–5.2)	**0.029**
Fasting blood glucose^b^	95.5 (84.0–102.0)	90.5 (84.0–99.8)	96.5 (85.8–103.0)	0.099
HOMA-IR^b^	3.4 (2.0–6.0)	2.4 (1.4–3.7)	4.8 (2.3–6.5)	**0.010**
Triacylglycerols^b^	148 (107–213.8)	133.5 (92.3–178)	194 (117–245.5)	**0.018**
Total cholesterol^a^	202.5 (37.8)	201.2 (43.7)	203.7 (31.4)	0.784
HDL-C^b^	45 (37.0–53.0)	43 (36.3–55.8)	46 (40.0–53.0)	0.907
LDL-C^a^	121.7 (31.9)	124.6 (35.3)	118.6 (28.1)	0.441
VLDL-C^b^	30 (21.0–40.8)	27.0 (18.3–35.8)	38.5 (23.0–46.5)	0.057
Non-HDL-C^a^	155.1 (35.0)	153.6 (39.9)	156.5 (29.6)	0.727
hs-CRP^b^	0.23 (0.11–0.55)	0.18 (0.10–0.45)	0.26 (0.16–0.66)	0.405
Systemic blood pressure^c^				
Normal	28 (38.9)	17 (47.2)	11 (30.6)	0.147
Elevated	44 (61.1)	19 (52.8)	25 (69.4)	

Significant values are in bold.

^a^Mean (standard deviation); ^b^median (1st quartile–3rd quartile); ^c^n (%); *NCD* noncommunicable disease, *HOMA-IR* Homeostatic Model Assessment of insulin resistance, *HDL-C* High-density lipoprotein cholesterol, *LDL-C* Low-density lipoprotein cholesterol, *VLDL-C* Very low-density lipoprotein cholesterol, *hs-CRP* C-reactive protein high-sensitivity, Waist circumference (n = 66); Visceral Adiposity Index (n = 68); HDL-C, Non-HDL-C e us-CRP (n = 71); LDL-C e VLDL-C (n = 70).

### Lipidomic analysis

**Figure 1:**
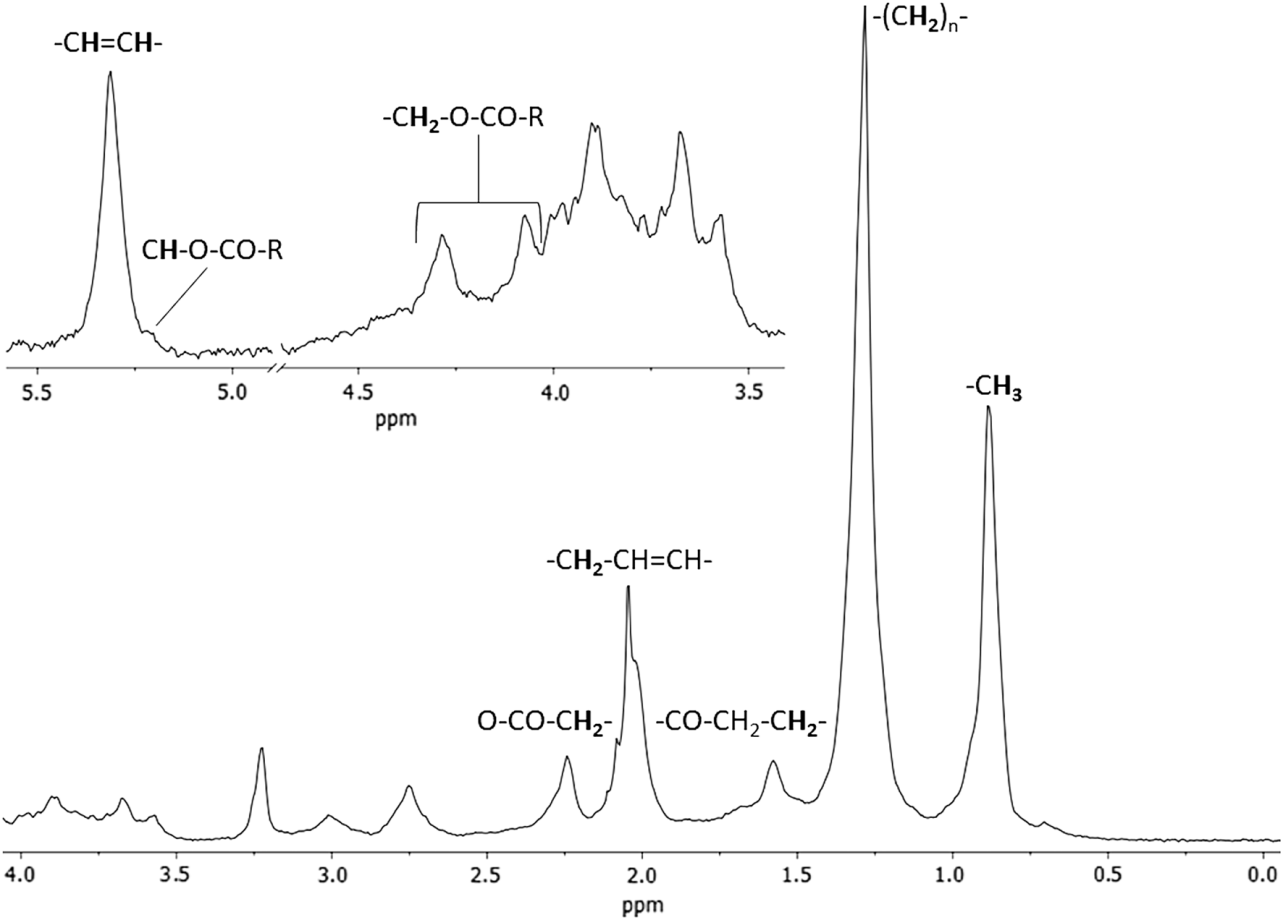
Example of the diffusion-edited ^1^H-NMR spectrum with assignments of the most important lipids for lipidomic analysis of two studied groups of individuals.

Data obtained by metabolomics using ^1^H-NMR NOESY 1d and CPMG spectra indicated a greater difference between the groups in the lipid class compounds, as shown in Supplementary Fig. S1. Therefore, we present and discuss the diffusion-edited ^1^H-NMR data below. Diffusion-edited ^1^H-NMR spectra (Fig. 1) showed characteristic peaks for low- and medium-polarity lipids, mostly attributed to saturated FA TAGs and, to a lesser extent, to unsaturated FA (–CH=CH–). Also, we provide an overlapping of diffusion-edited ^1^H-NMR spectra of all obese and non-obese individuals (see Supplementary Fig. S2). The assignments of all lipids are shown in Table 2.

**Table 2:** Attributions of lipids found as differentials between obese and non-obese individuals according to diffusion-edited ^1^H-NMR data.

Nº	Fragment of the corresponding molecule	δ ^1^H identified (ppm)
1	–C**H**_3_	0.50–1.10
	FA	0.5–0.8
	Cholesterol esterified	0.8–1.1
2	–(C**H**_**2**_)_n_– FA	1.20–1.30
3	–CO–CH_2_–C**H**_**2**_– Cholesterol and FA (TAG, Phospholipids)	1.50–1.60
4	–C**H**_**2**_–CH=CH– (Allylic)	1.95–2.01
5	O–CO–C**H**_**2**_– (FA)	2.20–2.30
6	–C**H**_**2**_–CO–O–R (sn3 + sn1)	4.00–4.40
7	–C**H**–CO–O–R (sn2)	5.15–5.25
8	–C**H**=C**H**– (olefinic, UFA)	5.25–5.40
9	–C**H**_2_–CH=CH– (UFA)	2.01–2.07
10	–OOC–C**H**_2_–CH_2_ (FA)	2.33
11	–CH=CH–C**H**_2_–CH=CH–	2.67–2.76
12	Glycerol in TAG (2′-CHOCO–)	5.26
13	PUFA (9-cis, 11-cis) H9, H12 (–C**H**=)	5.40
14	PUFA (10-cis, 12-cis) H10, H13 (–C**H**=)	6.13
15	PUFA (10-cis, 12-cis) H11 (–C**H**=)	6.22
16	PUFA (9-cis, 11-cis) H10, H11 (–C**H**=)	6.22
17	PUFA (10-cis, 12-cis) H11, H12 (–C**H**=)	6.85
18	Phospholipids	FA (CH_3_–, –CH_2_– and other PUFA)
19	Phosphatidylcholines	3.18–3.21 (C**H**_3_)_3_N^+^); FA; (CH_3_–, –CH_2_– and other PUFA)
20	Phosphorylcholine	3.21; 3.58; 4.17

*FA* Fatty acid, *UFA* unsaturated fatty acids, *PUFA* polyunsaturated fatty acids, *TAG* triacylglycerol.

Chemical shifts (^1^H-NMR) can be grouped into several defined regions: olefinic protons of single double bonds in the region of 6.20–5.30 ppm, glycerol protons of 5.10–3.70 ppm, the allylic protons of 3.05–2.60 ppm, the α-CH_2_ protons of 2.50–2.30 ppm, the CH_2_–CH=CH protons of ~ 2.0 ppm and the protons (CH_2_)_n_ and CH_3_ in the regions of 1.60–1.20 ppm and 0.98–0.86 ppm, respectively. It is important to highlight that the polar lipids of phospholipid classes, predominantly phosphatidylcholines, present chemical shifts superimposed with FA residues from TAG and even protons in regions of 3.18–3.21 ppm.

Principal component analysis (PCA) was performed. This analysis provides a rearrangement of the data, presenting them in a set of axes termed Principal Components (PC). This analysis allowed observation of the natural organization of the data^17^. PCA analysis resulted in a 2-component model, which explained 91.5% of the total variation in lipid metabolites between groups. PC 1 was responsible for 81.4% of the total variance analyzing the sample data according to the level of plasma lipids, while PC 2 was responsible for 10.1% (see Supplementary Fig. S3A). In addition to the PCA score plot, a loading plot of the PCA model provides information about which variables contribute to the components, as shown in Supplementary Fig. S4.

**Figure 2:**
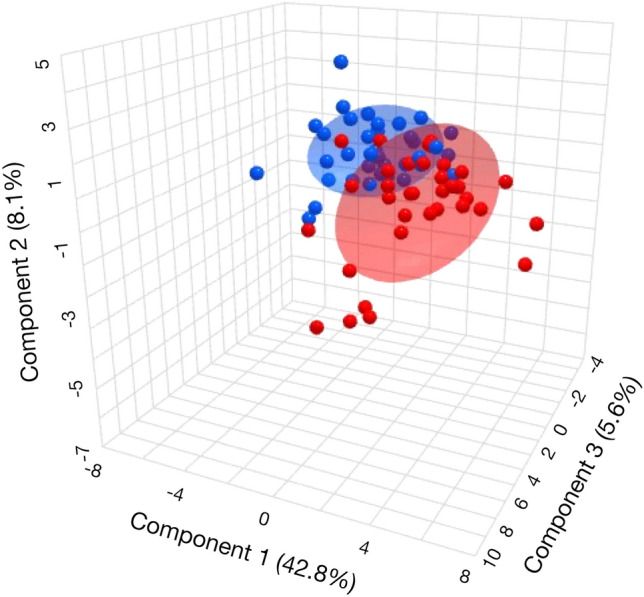
3D Score plot illustrating the PLS-DA model of two studied groups obese (BMI ≥ 30 kg/m^2^) (red) and non-obese (BMI < 30 kg/m^2^) (blue) individuals.

In addition, partial least squares discriminant analysis (PLS-DA) was applied to classify the samples as this method overcomes the limitations of other methods, allowing the handling of many variables^17^. 3D Score plot illustrating the PLS-DA model showed a greater dispersion in the group of individuals with obesity compared to non-obese (Fig. 2). We found that the PLS-DA model presented a result similar to that of the PCA since the two components were able to explain 91.3% of the total variation in lipid metabolites between the groups, with latent variable (VL) 1 being responsible for 80.5% of the variance and VL 2 10.8% (see Supplementary Fig. S3B). An orthogonal PLS-DA (OPLS-DA) model, a variant of the PLS-DA method, was also used (see Supplementary Fig. S3C). This method is based on orthogonal partial least squares discriminatory analysis, and the main difference between the methods is interpretability, since PLS-DA separates variability into systematic and residual, whereas OPLS-DA separates predictive, orthogonal, and residual variabilities^17^. The data obtained from OPLS-DA analysis showed clustering of samples with R^2^X, R^2^Y, and Q^2^ values of 21.5%, 21.4%, and 17.6%, respectively.

**Figure 3:**
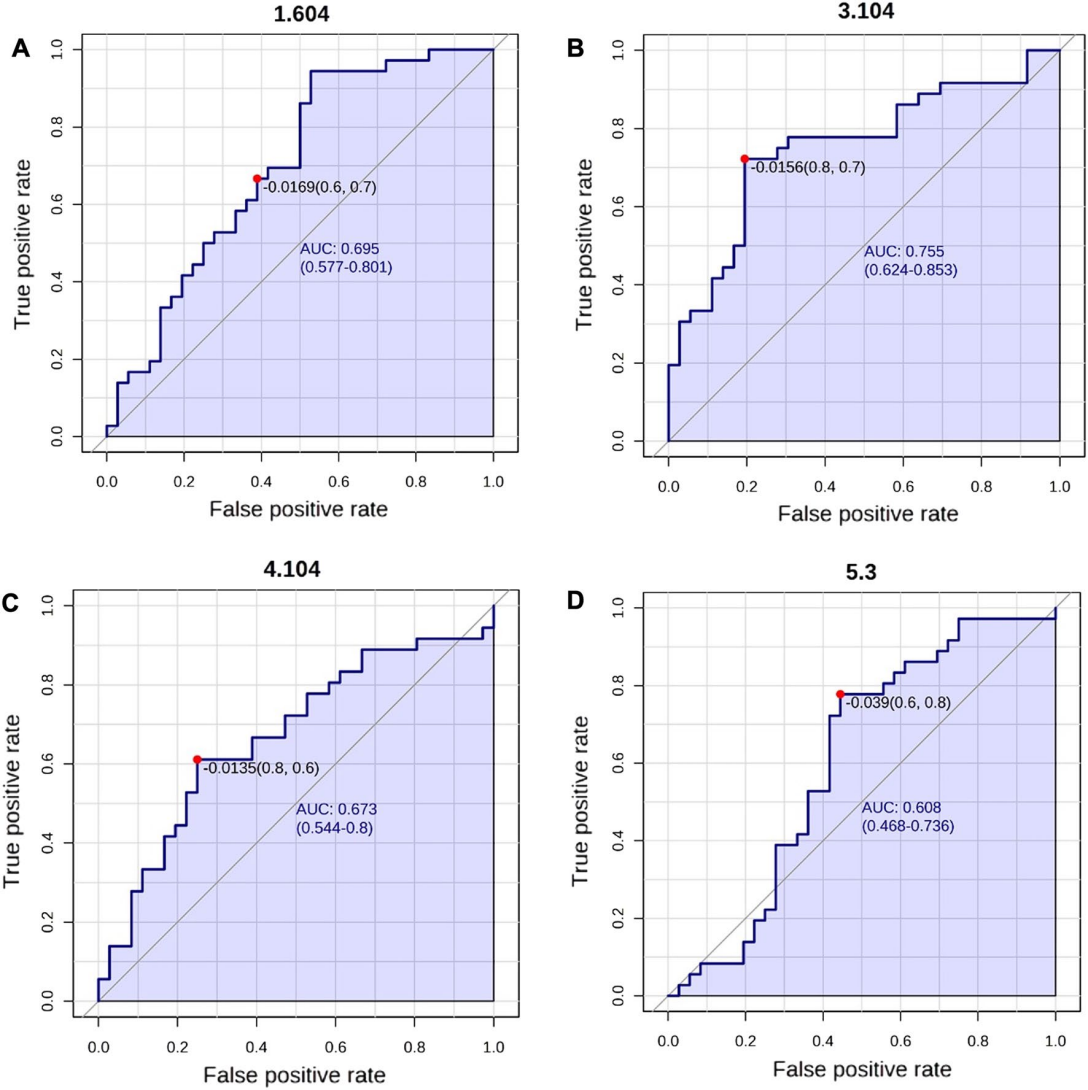
The receptor operation characteristic (ROC) curves for lipids' peaks (**A**–**D**) were obtained from the chemometrics data that distinguished the obese from non-obese individuals. The optimal cut-off point is represented on the curve with a red dot and a line on the box plot.

The area under the receiver operating characteristic (ROC) curve was constructed to assess the accuracy of the chemometrics models. The highest values of the area under the ROC curve were obtained for four peaks (Fig. 3), demonstrating a moderate accuracy (0.608 for 5.3 ppm to 0.755 for 3.104 ppm) of the PLS-DA model for discriminating patients.

**Figure 4:**
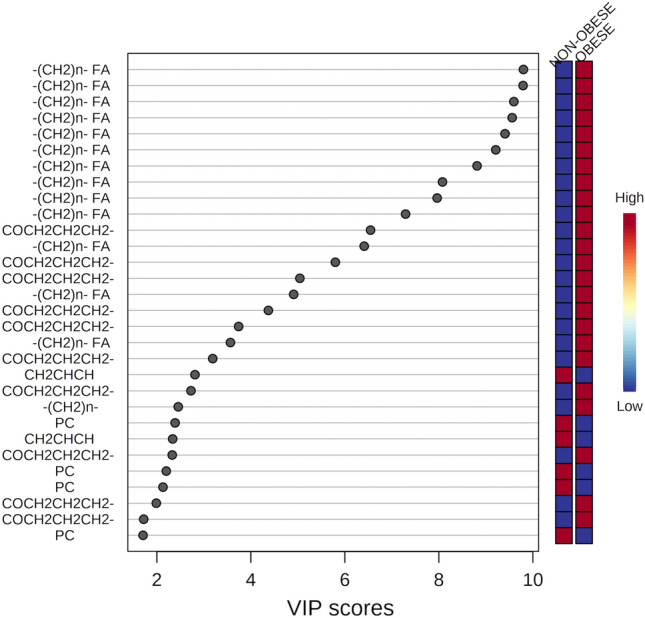
Graph illustrating thirty variables, i.e. chemical shifts, corresponding to assigned lipids with VIP values greater than 1.95 according to PLS-DA results. The colored scale on the right represents the variation in concentrations of lipids in the obese and non-obese groups.

Figure 4 presents the importance of the variables in the projection (VIP) of the model, considering the scores of lipid metabolites. As illustrated in Fig. 4, the variables with higher VIP scores were the most characteristic of the obese group. Among the lipids with the highest VIP values, which were predominantly SFAs, only six metabolites had lower concentrations in the obese group. These were PC, saturated [2-(CH_2_)_n_ FA], and unsaturated FA residues, with one unsaturation, corresponding to the metabolite identified as –CH_2_–CH=CH-A.

**Figure 5:**
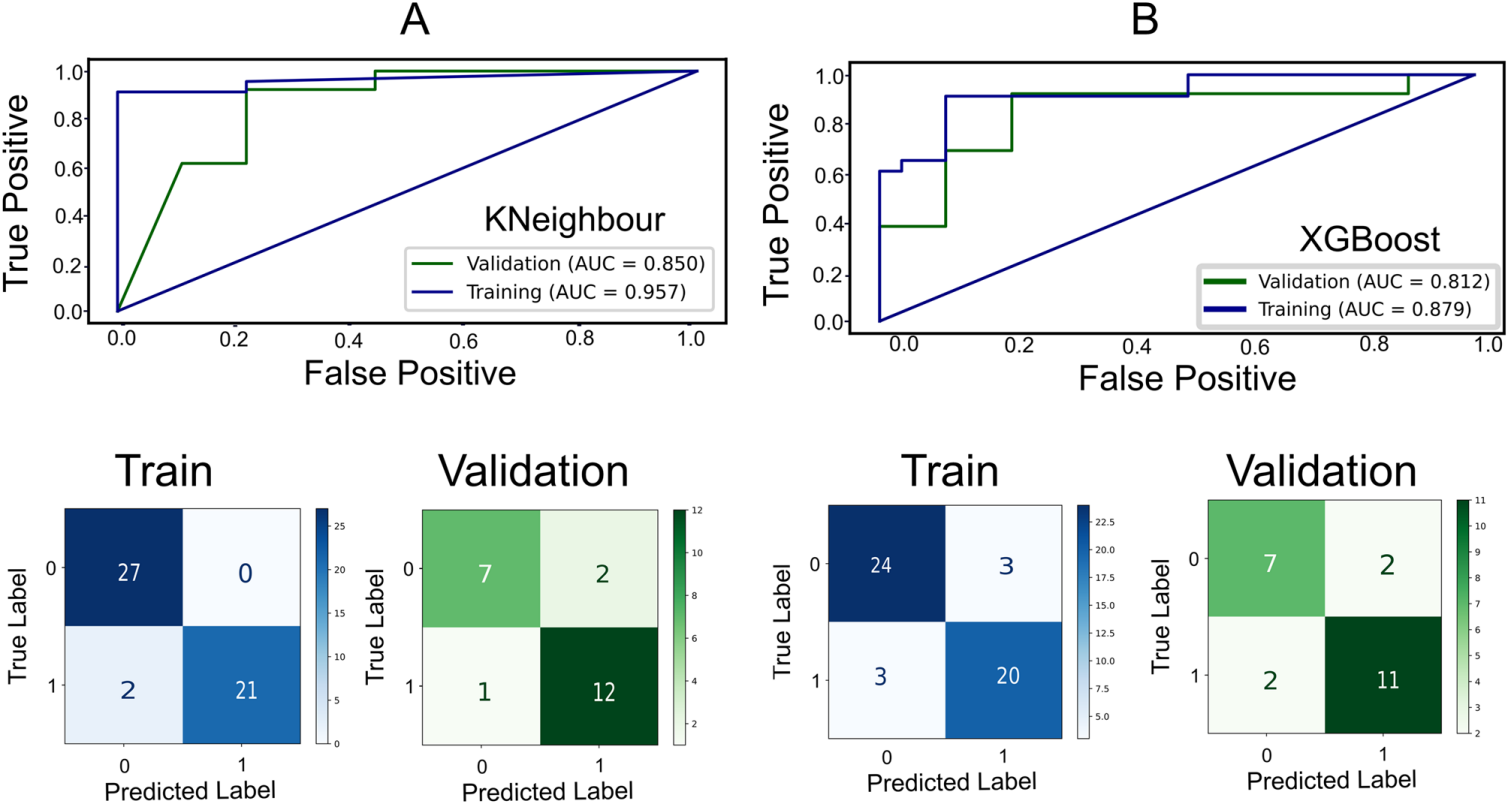
Cross-validation and test results and confusion matrix for train data and validation set (**A**) *k*-Neighbour (**B**) XGBoost.

Additionally, we applied ML techniques to classify obese and non-obese individuals based on selected NMR signals. We used two models: *k*-nearest neighbors (*k*NN) and XGBoost. The *k*NN model achieved an AUC of 0.96 for the training set and 0.85 for the validation set, indicating good performance in distinguishing between the two groups. The XGBoost model had a training AUC of 0.88 and a validation AUC of 0.81, also demonstrating good performance (Fig. 5). The models were generated using five non-co-correlating peaks from the NMR data. The signal at 1.50–1.60 ppm (–CO–CH_2_–CH_2_–, Cholesterol and FA–TAG, Phospholipids) showed the highest importance in both models (Table 3). The second priority signal was identified at 4.00–4.40 ppm. Among all peaks, 5.15–5.25 and 5.25–5.40 ppm showed the least importance. In terms of performance metrics, the *k*NN model had an accuracy of 0.86, balanced accuracy of 0.85, precision of 0.86, *F*1 score of 0.89, recall of 0.92, and ROC-AUC of 0.85. The XGBoost model demonstrated a balanced accuracy of 0.81, accuracy of 0.82, precision of 0.85, *F*1 score of 0.85, recall of 0.85, and ROC-AUC of 0.81.

**Table 3:** Feature importance score in percentage for *k*-neighbour and XGBoost Model.

NMR peaks	Importance %	
	k-neighbour	XGBoost
1.50–1.60 ppm: –CO–CH_2_–C**H**_**2**_–, Cholesterol and FA (TAG, Phospholipids)	30	59
3.18–3.21 ppm: Phosphatidylcholines, (C**H**_3_)_3_N^+^); FA; (CH_3_–, –CH_2_– and other PUFA)	15	2
4.00–4.40 ppm: –C**H**_2_–CO–O–R (sn3 + sn1)	32	34
5.15–5.25 ppm: –C**H**–CO–O–R (sn2)	13	0
5.25–5.40 ppm: –C**H**=C**H**– (olefinic, UFA)	10	5

**Figure 6:**
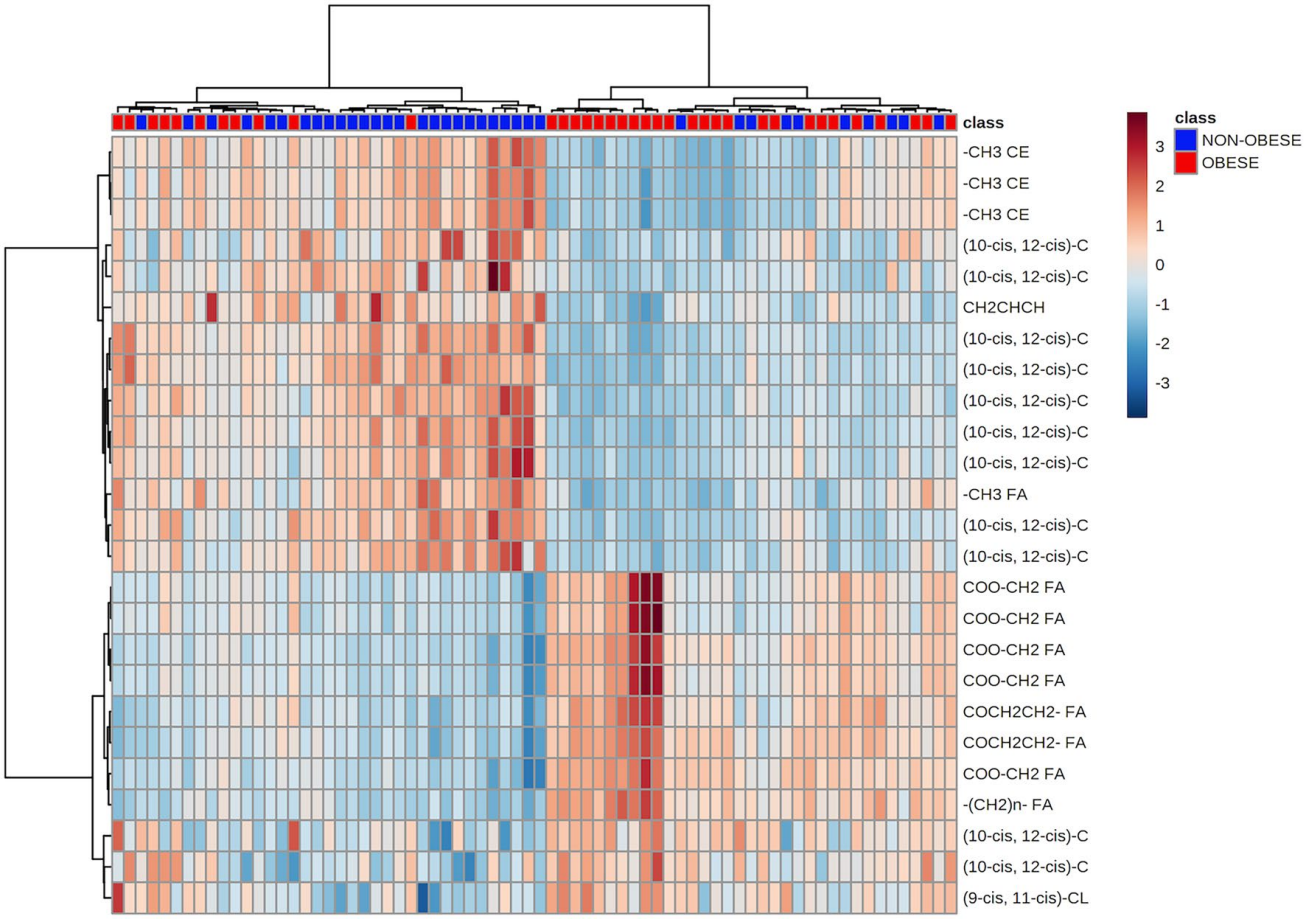
The clustering result is shown as a heatmap (distance measurement using Euclidean and clustering algorithm using ward.D). Obese individuals are marked in red.

A hierarchical cluster analysis was also performed, which verified whether sets of variables were causally linked to each other or showed relationships that constituted the clusters. These clusters were then constructed hierarchically so that the two closest clusters were merged into the same cluster (Fig. 6). It is important to highlight that the obese group (BMI ≥ 30 kg/m^2^) showed a lower amount of PUFAs in the lipid profile, as can be seen in the light-blue quadrant with a concentration of 14 metabolites. The results of this analysis showed that 11 individuals in the group without obesity showed similarities in lipid profiles to the group of individuals with obesity. Thus, it is extremely important to pay attention to the metabolic profile of 1/3 of individuals without obesity who, despite having a BMI < 30 kg/m^2^, may present changes in the profile of lipid metabolites and risk factors for cardiovascular disease.

## Discussion

To our knowledge, this was the first study to investigate the lipidomic profiles of adults and older people in a city in northeast Brazil. The main findings were the higher number of obese women and notable variations in cardiometabolic risk factors, such as hypertriglyceridemia, insulin resistance, increased WC, and VAI. We observed a trend towards higher concentrations of lipid metabolites in the obese group, with a predominance of saturated lipid metabolites and lower levels of unsaturated lipids. In contrast, some species of PC were lower in the group of individuals with BMI ≥ 30 kg/m^2^.

### Cardiometabolic profile of obese and non-obese groups

This study revealed a cardiometabolic risk profile in obese individuals with an emphasis on a higher percentage of dyslipidemias, characterized by increases in TAG concentrations and reduction in HDL-C, in agreement with other findings in the literature^5,18^. Recurrent hypertriglyceridemia probably occurs in obese individuals as a consequence of increased insulin circulation, which generally stimulates the degradation of TAG-rich lipoproteins, while simultaneously delaying the plasma clearance of these lipoproteins, resulting in increased triglyceridemia^5^.

The obese population also had a higher WC and VAI, which are indicators of visceral fat accumulation. This metabolic profile of obese individuals directly contributes to hyperinsulinemia, systemic inflammation, and dyslipidemia^19^. In contrast to the findings of other studies, we did not observe any differences in the C-reactive protein high-sensitivity (hs-CRP) values between the groups in our study, which limited our interpretation of the actual inflammatory profile of obese individuals. The analysis of other inflammatory markers may provide more information about the chronic and low-grade systemic inflammation characteristic of obesity since other authors have already found a relationship between tumor necrosis factor-α (TNF-α), adipokines, and interleukin-6 and BMI^20^.

Another key finding of our study was the increase in HOMA-IR score in the obese group. HOMA-IR is an important indicator of insulin resistance, and its elevation indicates a greater risk of the development of type 2 diabetes mellitus (T2DM), which is the primary triggering factor of dyslipidemias^5^. Several prior studies have reinforced that both insulin resistance and dyslipidemia are related to structural and functional changes in adipose tissue^21,22^. These alterations trigger cascading mechanisms, such as endoplasmic reticulum stress and adipocyte mitochondrial dysfunction, the release of adipokines, and increased lipolysis, with increased release of free FAs and consequent lipotoxicity^23,24^. These mechanisms initiate the systemic inflammatory process characteristic of obesity, characterized by a low-intensity chronic reaction, so-called metabolic inflammation^25^.

### Lipidomic profile of obese and non-obese groups

Regarding our lipidomic profile results, we observed that lipid metabolites explained more than 90% of the variation between the two groups with a trend towards a higher concentration of these metabolites in the obese group. A similar result was observed in another study, in which 18 groups of individual lipid molecules explained 92.43% of the total variance among the three groups (eutrophic, overweight, and obese individuals)^26^. It has also been shown that the plasma lipidome is strongly associated with BMI, suggesting that lipid metabolites could potentially identify individuals who are overweight and probably also have cardiometabolic risk factor alterations^27^.

It is important to emphasize the characteristics of these lipid species that contribute to the differences between groups. In our study, the subjects with obesity had a predominance of saturated lipid metabolites, lower concentrations of unsaturated lipids, and some species of PC. These findings require attention, especially in obese individuals, considering that the degree of saturation of lipid species, especially those that are part of the cell membranes, can influence cell signaling events^28^. For example, researchers have previously pointed out that SFAs, especially lauric and palmitic SFAs, are capable of stimulating an inflammatory response via the toll-like receptors (TLR) 4 and TLR2 signaling pathways, and can impair insulin signaling and/or provide substrates for the synthesis of potentially harmful lipids, such as PL^29^.

However, past studies have suggested that PUFAs may impair lipopolysaccharide mediated TLR4 activation mechanisms, decreasing the activation of nuclear factor kappa B and the production of proinflammatory cytokines^28^. Additionally, several studies have shown that several polyunsaturated lipid metabolites (18:2) are negatively associated with BMI and WC, independent of baseline dietary intake^13^. A prior study further proposed several possible explanations for this association: (1) improvements in cell membrane fluidity and functions by PUFAs collectively improve insulin sensitivity, which is directly related to adipose tissue dysfunction; and (2) improved regulation of gene expression that controls fat oxidation and synthesis, such as sterol regulatory element-binding protein 1^30^.

Regarding MUFAs, a prior study identified that a diet rich in this type of fat decreased saturated diacylglycerol concentrations (16:0) and increased the proportion of unsaturated TAGs (22:1), indicating that the replacement of SFAs by MUFAs reduces the cardiometabolic risk^9^. The effects of this imbalance in FA intake observed in the present study seem to be more pronounced in individuals with obesity, which may have consequences on this lipidomic profile characterized by higher concentrations of saturated and less unsaturated lipid metabolites, which may be related to the metabolic changes already associated with greater visceral adiposity, insulin resistance, and hypertriglyceridemia.

With respect to PC, prior studies have identified findings similar to ours, observing an inverse relationship between some PC species and BMI. A possible explanation for this is the increased degradation of these species in obese individuals^14,31^. This degradation is related to proteins anchored to glycosylphosphatidylinositol (GPI-AP), which are released into circulation due to obesity. In a compensatory mechanism to balance the deleterious effect of circulating GPI-AP, these proteins are degraded, and consequently, a similar increase in lipolytic degradation could be observed because PCs are associated with GPI-AP in micellar complexes^32^. In addition, recent evidence indicates that glycerophospholipids are involved in essential signaling processes, highlighting the potential utility of this class of lipids as novel biomarkers of cardiometabolic risk^31^.

An interesting finding of our study was that 10 of the individuals without obesity had a lipidomic profile like that of obese individuals. By analyzing their data individually, these patients were characterized as being overweight (BMI > 24.9 kg/m^2^), 90% had dyslipidemia, and 80% had high adiposity and high cardiovascular risk according to VAI and WC, respectively. Therefore, regardless of obesity, these individuals showed a cardiometabolic risk of developing obesity-induced complications like those with obesity. It is worth noting that the analysis of BMI alone seems to no longer be sufficient to indicate cardiometabolic risk, and the distribution of body fat seems to be also associated with complications. For example, excessive visceral fat deposition is a key phenotype associated with arterial hypertension, dyslipidemia, and impaired glucose metabolism, which confer an increased risk for T2DM and cardiovascular diseases (CVD)^33^. As such, our results indicate that lipidomic profiling of these individuals without obesity will allow the identification of early cardiometabolic risk, probably associated with the distribution of body fat.

In addition, 11 obese individuals had a lipidomic profile similar to that of non-obese. The phenotypes of these individuals were heterogeneous; some did not have dyslipidemia but had other comorbidities, such as arterial hypertension and T2DM, while others had dyslipidemia but did not have any established metabolic syndrome. These individuals could be labeled as metabolically healthy obese (MHO); however, there is still no universal definition for this group of people^34^. Although it has been well defined in the literature that there are people with obesity who do not present metabolic and cardiovascular complications, the heterogeneous definitions of MHO are a limitation for the interpretation of studies that report associations between MHO, CVD, mortality, and risk for cardiometabolic diseases^33^. Analysis of the lipidomics profile of individuals with obesity could represent a method to identify whether subjects can be classified as MHO or if the cardiometabolic risk is already present.

Weight gain and the manifestation of cardiometabolic risk factors, such as high visceral adiposity, may be influenced by different factors, such as genetics, environment, and lifestyle^13^. Our study demonstrated that the lipidomic profile can identify variations in these factors, and therefore, can be useful for identifying individuals at higher risk. As such, it is essential to help health professionals make early and informed decisions about appropriate interventions.

The results of this study indicate the potential of using NMR signals for classifying obese and non-obese patients. While the graphs in Fig. 2 did not exhibit striking differences between the two groups, the greater dispersion observed in the obese group, particularly in the OPLS-DA model, suggests metabolic alterations associated with obesity. The AUC value of 0.78 for lipid metabolites in the ROC curve analysis demonstrates moderate accuracy in discriminating individuals with and without obesity.

The ML models achieved high accuracies in classifying individuals based on the selected NMR peaks. These results suggest that the NMR peaks, particularly the identified signals at 1.50–1.60 ppm, hold valuable information for distinguishing between obese and non-obese individuals. The performance metrics of the models, including accuracy, precision, *F*1 score, recall, and ROC-AUC, further emphasize their classification potential. Both models exhibited high accuracy rates, indicating their ability to correctly classify a significant portion of both obese and non-obese patients. The precision scores indicate a low rate of false positives, suggesting that when the models classified an individual as obese, they were often correct. The F1 scores reflect a balanced performance between precision and recall, considering both false positives and false negatives. The recall scores demonstrate high rates of true positives, indicating that the models effectively identified obese patients. The ROC-AUC values confirm the models' ability to discriminate between the two groups. These findings highlight the promising potential of NMR-based lipidomics coupled with ML in the classification of obese and non-obese patients. The identified NMR signals capture metabolic differences associated with obesity, and the ML models effectively leverage this information for accurate classification. Further research, including validation studies with larger and more diverse individual cohorts, is warranted to confirm the reliability and reproducibility of these results. If validated, this approach could have significant implications for obesity diagnosis and monitoring, providing a non-invasive and potentially efficient tool for healthcare practitioners.

### Study limitations

This study had some limitations, such as the lack of identification of individual lipid species, making comparisons with other studies difficult. In addition, this study was performed on only a small portion of the population residing in a city in northeast Brazil; as such, the results should be generalized with caution. In addition, we did not explore data on food intake, but we also need to consider that it is not unusual for individuals with obesity to underreport their usual dietary intake intentionally or nonintentionally^34^, so data on food intake, mainly for the obese group, might be underestimated. Another limitation was the exclusive use of hs-CRP to assess the inflammatory profile since this marker can be altered due to other reasons; the use of markers such as TNF-alpha, interleukins, or adipokines would be a better option to provide a view of the inflammation related to obesity. Also, information about the use of medicines that could alter lipid metabolism was unavailable. On the other hand, this was the first study to evaluate the lipidome of the population in question and identify that it is related to risk factors, such as adiposity and insulin resistance. Another strength was the matching of the study population by sex and age, which reduced the chance of interpretation bias.

## Conclusion

In conclusion, the cardiovascular risk profile characterized by high WC, VAI, hypertriglyceridemia, and insulin resistance were identified in individuals at risk of obesity-induced metabolic complications. Probably, the imbalance in dietary FAs has significant repercussions in individuals with obesity, whose ^1^H-NMR lipidomics results showed higher SFA-rich lipids and lower PUFA species. Our findings highlight the value of lipidomics in predicting cardiovascular risk in overweight individuals, which may lead to early intervention in clinical practice. However, it is also important to consider the effects of diet on cardiometabolic risk phenotype. We hope that this study furthers the literature on this topic, ultimately improving our understanding of the pathophysiological mechanisms that lead to the development of metabolic complications resulting from obesity.

## Methods

### Participant recruitment and ethics

This cross-sectional study was approved by the Research Ethics Committee of the Federal University of Rio Grande do Norte (CAAE number 96294718.4.2001.5292) in accordance with the Brazilian guidelines for research involving human beings. Eligible participants were informed about the objectives, risks, and benefits. Informed consent was obtained from all subjects and/or their legal guardian(s).

The participants included in this study were recruited from the Brazilian Usual Consumption Assessment (BRAZUCA), and the sampling plan considered a probabilistic sample by clusters in two stages (census sectors and households). The sample was composed of adults (20–59 years old) and older people (60 years old or more) of both sexes, residing in the city of Natal, northeast Brazil. The exclusion criteria were: pregnant and lactating women, users of illicit drugs, people who underwent chemotherapy and/or radiotherapy in the last 6 months, and those who were unable to answer the research questions. Recruitment performed between June 2019 and March 2020 resulted in a subsample of 112 participants whose biological samples were withdrawn. A sample size calculation for this study showed 172 participants, 86 in each group, considering an effect size d 0.5, α error 0.05, and power of 90%. Unfortunately, the data collection was interrupted due to a public health emergency of international importance arising from the COVID-19 outbreak. Thus, the final sample size of this study included 72 participants, perfectly matched by age and sex, to reduce the probability of bias errors divided into two groups: obese individuals (n = 36, BMI ≥ 30 kg/m^2^) and non-obese individuals (n = 36, BMI < 30 kg/m^2^). This final sample had a statistical power of 55%.

### Data collection

Sample collection was performed in participants’ residences with a standardized and revised questionnaire designed in accordance with the protocols of the National Health Survey. The questionnaire included questions on socio-demographic data, lifestyle (smoking and alcohol consumption), and noncommunicable disease diagnoses (self-reported chronic diseases such as diabetes mellitus and arterial hypertension). Based on this information, the global risk score was calculated, as proposed by the Brazilian Dyslipidemia Guideline^35^. Anthropometric measurements such as weight and height were measured, and BMI was classified according to the cut-off points recommended by the World Health Organization (WHO)^1^. We assessed WC at the midpoint between the last rib and the iliac crest using an inextensible measuring tape, and classification considered cut-off points proposed by WHO^1^. We also measured the blood pressure using a mercury column blood pressure plus device (SKU 001 model, Unitec^®^, São Paulo, SP, Brazil), as recommended by the Brazilian Guidelines on Arterial Hypertension—2020^36^. Blood samples were also collected to evaluate metabolomic data, lipid profile (total cholesterol, LDL-C, HDL-C, non-HDL-c, and TAG), glycemic profile (fasting glucose and fasting insulin), and hs-CRP. Additionally, the VAI was calculated. This is a sex-specific mathematical model based on anthropometric measurements (BMI and WC), and functional parameters (TAG and high-density lipoprotein (HDL) cholesterol), which indicate adipose tissue dysfunction. We considered the cut-off points proposed by Amato and Giordano (2014)^37^.

### Biochemical analyses

Blood samples (10 mL) were collected in the morning after an overnight fast (12 h). Fasting blood glucose, total cholesterol, and TAG levels were assessed using the enzymatic method, and HDL-c levels were measured by homogeneous colorimetric methods. Low-density lipoprotein (LDL-c) values were determined using the Friedewald formula [LDL-c = total cholesterol − HDL-c + (TAG/5)]. We also calculated the non-high-density lipoprotein cholesterol (non-HDL-c) based on the difference between total cholesterol and HDL-c^38^. We performed an immunoassay using the sandwich technique for insulin level determination. The hs-CRP was analyzed by immunoturbidimetry. All analyses were performed in an automated manner (COBAS 6000; Roche^®^ Professional Diagnostics, Risch-Rotkreuz, Suíça). We used the homeostasis model assessment—insulin resistance (HOMA-IR) index to evaluate insulin resistance. The values were obtained using insulin fasting values and fasting blood glucose and considered the cutoff point > 2.7 as indicative of insulin resistance^39^. Metabolic syndrome was diagnosed according to the 2009 ‘harmonized’ criteria^40^, which qualify a person with metabolic syndrome through the presence of at least three of five criteria, which are: abdominal circumference > 102 cm in men or > 88 cm in women; fasting glucose ≥ 100 mg/dL or drug treatment for diabetes; TAG ≥ 150 mg/dL; HDL-c < 40 mg/dL in men or < 50 mg/dL in women; systolic blood pressure ≥ 130 mmHg or diastolic ≥ 85 mmHg or under drug treatment for hypertension.

### ^1^H-NMR analyses

Blood plasma was carefully identified and stored at − 80 °C and used to evaluate metabolomic data. The samples (500 μL) were then thawed on ice and further mixed with 100 μL of deuterium oxide (D_2_O 99.9%, Cambridge Isotope Laboratories, Inc., Andover, USA) at room temperature, centrifuged at 12,000×*g* for 2 min at 4 °C, and placed into 5 mm NMR tubes. High-resolution ^1^H-NMR (noesypr1d), T_2_-edited (cpmgpr1d), and diffusion-edited (stebpgp1s191d) spectra were acquired using a Bruker AVANCE III 600 MHz spectrometer with a triple-inverse probe (TBI) at 25 °C. Total correlation spectroscopy (TOCSY) spectral data were recorded using the mlevphpr pulse sequence, and single heteronuclear quantum correlation (HSQC) spectral data were obtained with the hsqcedetgpsp.3 pulse sequence. Scalar coupling was further evaluated from J-RESolved Spectroscopic Experiments (JRES) using the jresgpprqf pulse sequence. 1D and 2D NMR data, such as chemical shifts, coupling constants, the multiplicity of peaks, and metabolomic databases, in agreement with the Human Metabolome Database (HMDB) and BioMagResBank (BMRB)^41,42^, were used for metabolite assignments.

### Statistical analysis

Kolmogorov–Smirnov test was performed, and variables identified as parametric were described as the mean (standard deviation), nonparametric variables as median (interquartile range), and categorical variables as absolute and relative frequency. To compare the categorical variables between the two groups, the chi-square test was performed, and the numerical variables were compared using Student’s t-test or Mann–Whitney U test, where parameters were not normally distributed.

Plasma ^1^H-NMR spectra were separated according to the type of experiment performed (NOESY, CPMG, or edited by diffusion) and processed as follows: baseline and phase were corrected, and spectra were aligned and referenced (lactate signal, 3H, δ 1.33,* J* = 7.0 Hz, doublet) in MestreNova Inc. 14.2 software. Individuals with a BMI ≥ 30 kg/m^2^ were classified as “obese” and were compared with those with a BMI < 30 kg/m^2^, classified as “non-obese”. The individuals were matched by age and sex. The spectra were normalized by median and mean centering, and the water peak was excluded (HDO signal, 4.65–5.20 ppm, singlet), binned (0.005 ppm), and exported in the data.csv format for chemometrics analysis. Chemometric analyses were performed using MetaboAnalyst 5.0 software platform (https://www.metaboanalyst.ca/)^43^.

The lipid intensity data after normalization to a log format at base 10 were subjected to PCA for all samples to explore inherent clusters in the data and identify outliers. Data were modeled using the supervised OPLS-DA method to identify differences in metabolites between the groups. Variable Importance in projection (VIP) scores were estimated using the PLS-DA model^44^. For all analyses, values with p < 0.05 were considered statistically significant.

### Machine learning models

We carried out a binary classification on the NMR peaks using *k-*nearest neighbors (*k*NN) and XGBoost (XGB) to classify obese and non-obese groups. The data matrices were split into training (70% of the data) and validation (30% of the data). In the *k*NN method, the most common class between the *k*-nearest neighbor was determined. The extreme gradient boosting algorithm (XGBoost) is an ensemble ML model that uses the gradient boosting algorithm during the classification of obese and non-obese providing parallel tree boosting. To assess the diagnostic value of the NMR signals, we constructed ROC curves. The area under the ROC curve (AUC) was calculated to determine the accuracy of the model in discriminating between obese and non-obese individuals. The metrics used for the evaluation of the models were accuracy, *F*1-score recall (sensitivity), and specificity. To get a clear understanding of the model’s performance, we prioritized the analysis of the areas under the curve (ROC-AUC).

$$\mathrm{Accuracy }= (TC + TN) / (TC + TN + FC + FN),$$

$$\mathrm{Recall }= TC / (TC + FN),$$

$$\mathrm{Precision }= TC / (TC + FC).$$

In the above formulas, *TC* represents the correct classification between the Obese and Non-obese group, *FC* represents the incorrect classification of obesity, *TN* represents the correct classification of normal subjects, and *FN* represents the incorrect classification of normal subjects.

### Equipment and settings

To generate the ^1^H-NMR data figures and signal assignments (Fig. 1 and Supplementary Fig. S2) MestreNova Inc. 14.2 software and Topspin software were used. For the statistical model figures (Figs. 2, 3, 4, 5 and 6, and Supplementary Figs. S1, S3 and S4), the MetaboAnalyst platform and Python (version 3.8.12) was used.

## Supplementary Information

Supplementary Figures.

## Data Availability

All data generated or analyzed during this study are included in this manuscript and its supplementary information file. Further inquiries can be directed to the corresponding author.
