# Supplementary material for: Plasma lipid metabolites as potential biomarkers for identifying individuals at risk of obesity-induced metabolic complications
Source: Sci Rep. 2023 Jul 20;13:11729. doi: 10.1038/s41598-023-38703-8 (PMC10359283; doi:10.1038/s41598-023-38703-8)
Supplement: Supplementary file 1 — Supplementary Figures. [file 41598_2023_38703_MOESM1_ESM.pdf]

Plasma lipid metabolite as potential biomarkers for identifying individuals at risk of obesity-induced metabolic complications

Paula Emília Nunes Ribeiro Bellot[1], Erik Sobrinho Braga[2], Folorunsho Bright Omage[2,3], Francisca Leide da Silva Nunes[1], Severina Carla Vieira Cunha Lima[4], Clélia Oliveira Lyra[4], Dirce Maria Lobo Marchioni[5], Lucia Fatima Campos Pedrosa[4], Fernando Barbosa Jr.[6], Ljubica Tasic[2], Karine Cavalcanti Maurício Sena-Evangelista[4]

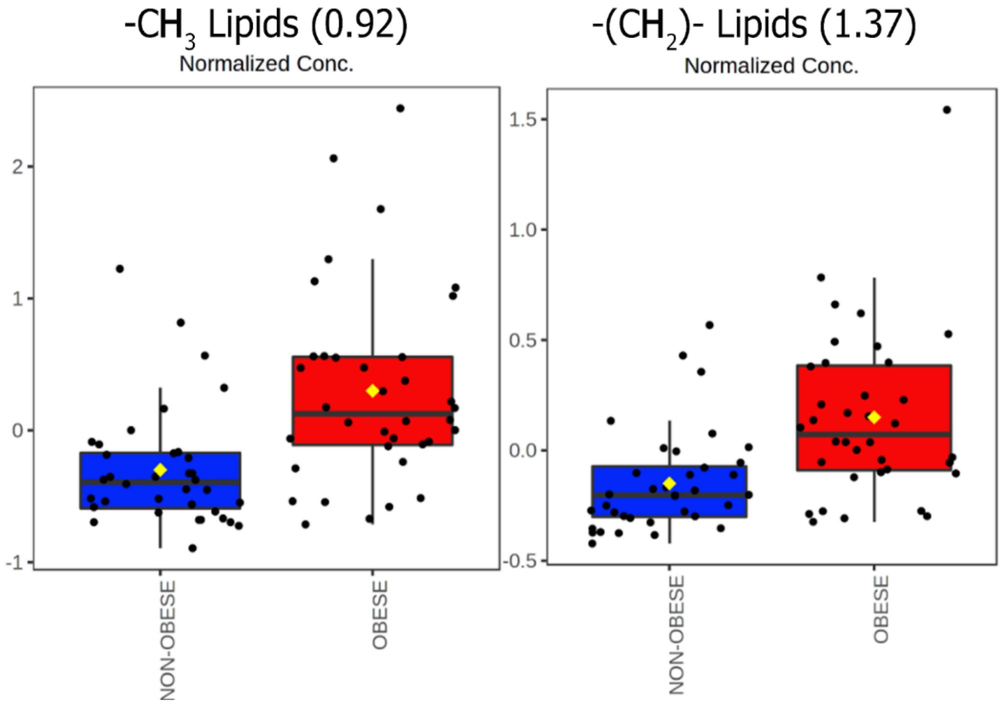

Figure S1. Boxplots of the main plasma lipids were found to be discriminatory between obese (red boxes) and non-obese patients (blue boxes). Each box contains the lipid variations according to a t-test univariate analysis.

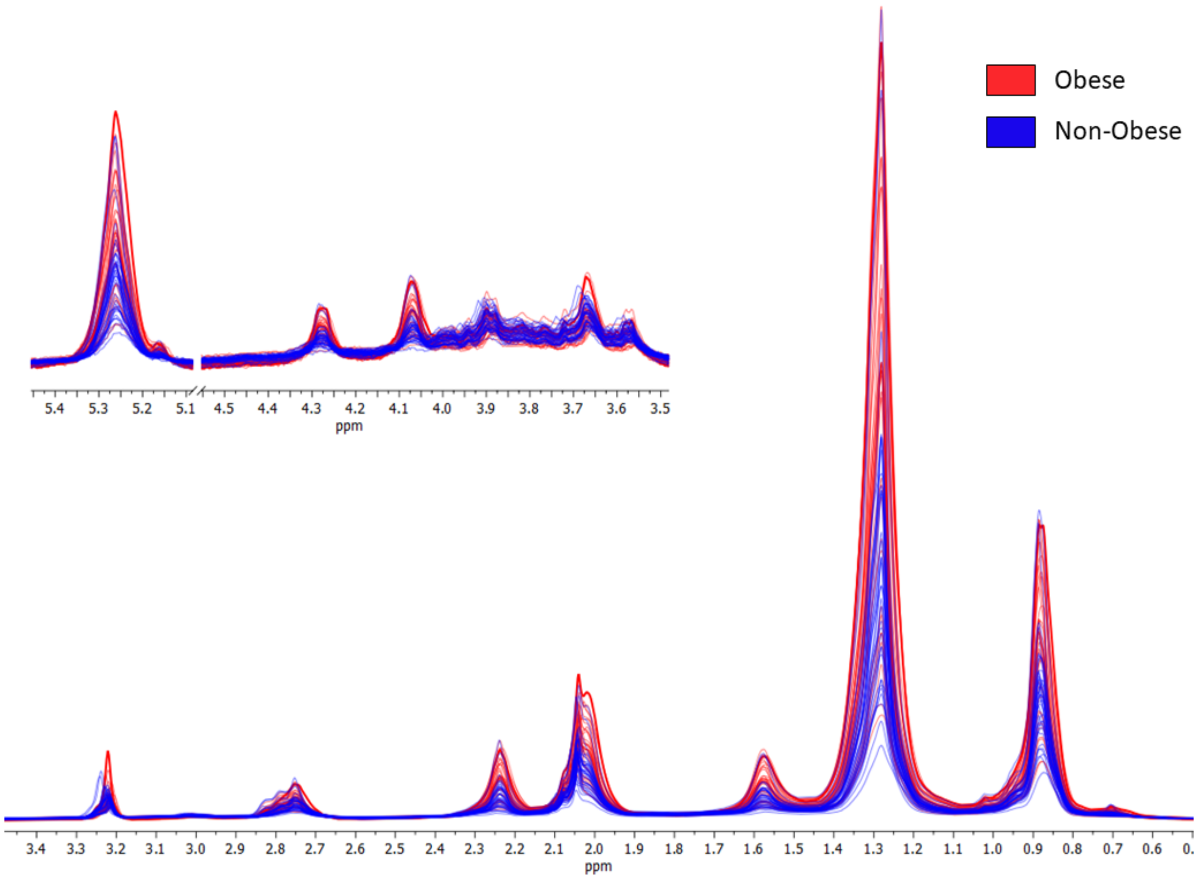

Figure S2. Overlapping diffusion-edited ^{1}H-NMR spectra of obese (red line) and non-obese (blue line) groups of all investigated samples.

A.

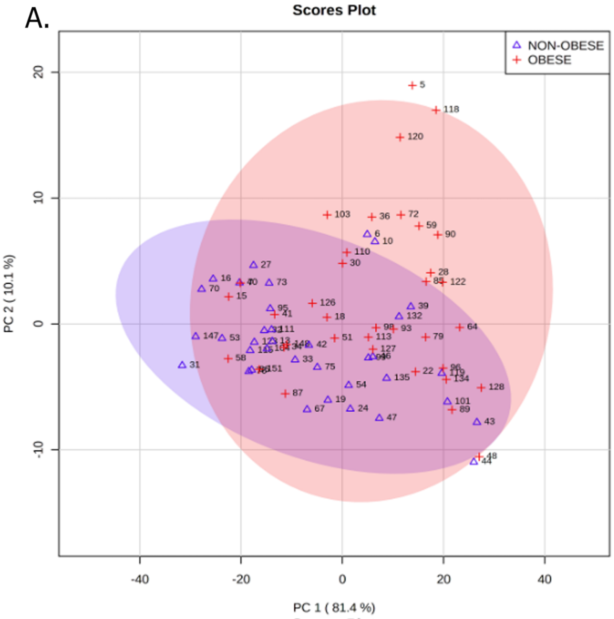

Scores Plot
NON-OBESE
OBESE
PC 2 (10.1 %)
PC 1 (81.4 %)

B.

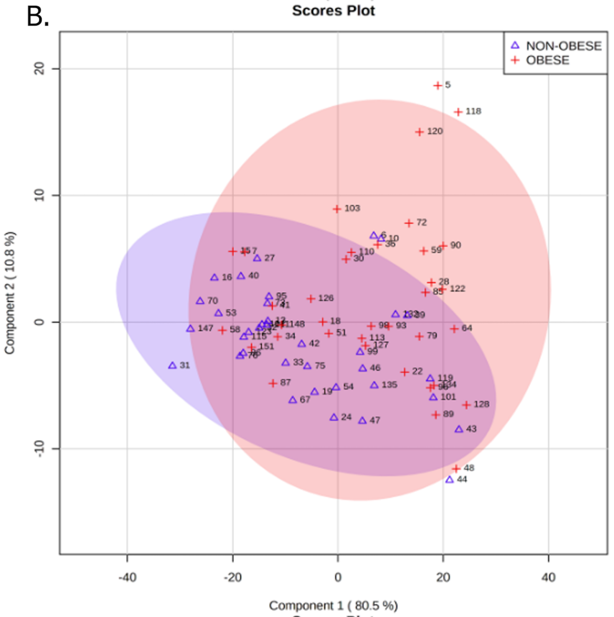

Scores Plot
NON-OBESE
OBESE
Component 2 (10.8 %)
Component 1 (80.5 %)

C.

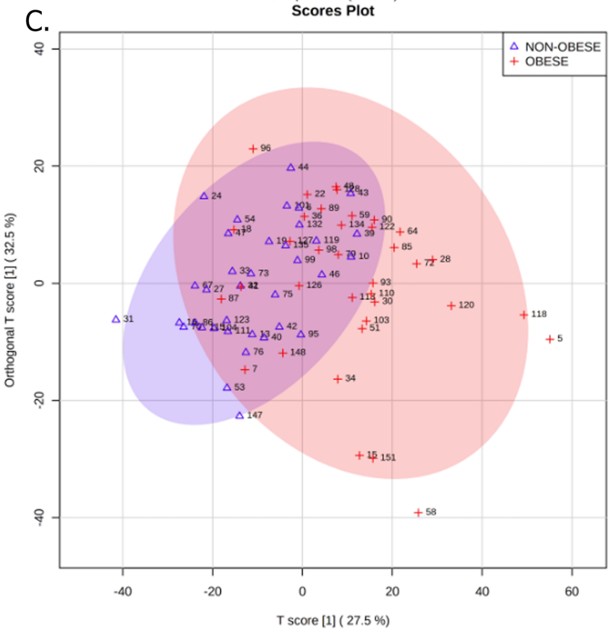

Scores Plot
NON-OBESE
OBESE
Orthogonal T score [1] (32.5 %)
T score [1] (27.5 %)

Figure S3. Score plots illustrating the two studied groups obese (BMI ≥ 30 kg/m^2) (red triangles) and non-obese (BMI < 30 kg/m^2) (blue cross) individuals. **(A)** 2D PCA model. **(B)** 2D PLS-DA model. **(C)** 2D OPLS-DA Model

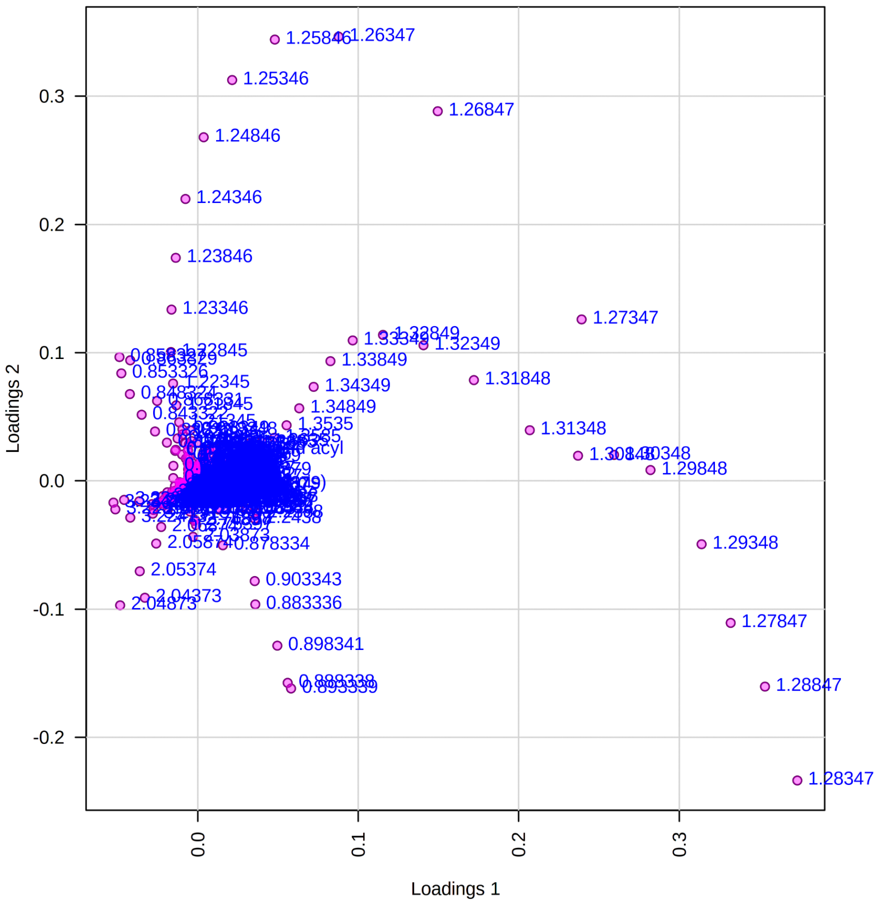

Figure S4. Loading plot of the PCA model. The loading plot provides information about which variables contribute to the components. Values towards 1 or -1 describe that the variable strongly influences the component. Values close to 0 indicate that the variable has a weak influence on the component. The sign of a loading (+ or -) indicates whether a variable and a principal component are positively or negatively correlated.

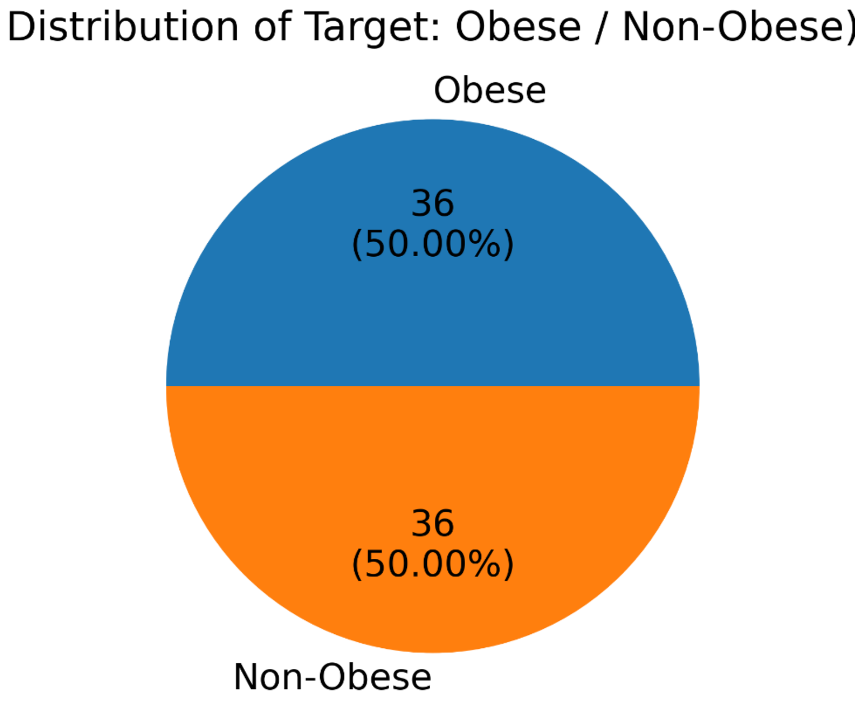

Figure S5: Distribution of the target feature, providing insights into the frequency or occurrence of the two classes within the target variable.

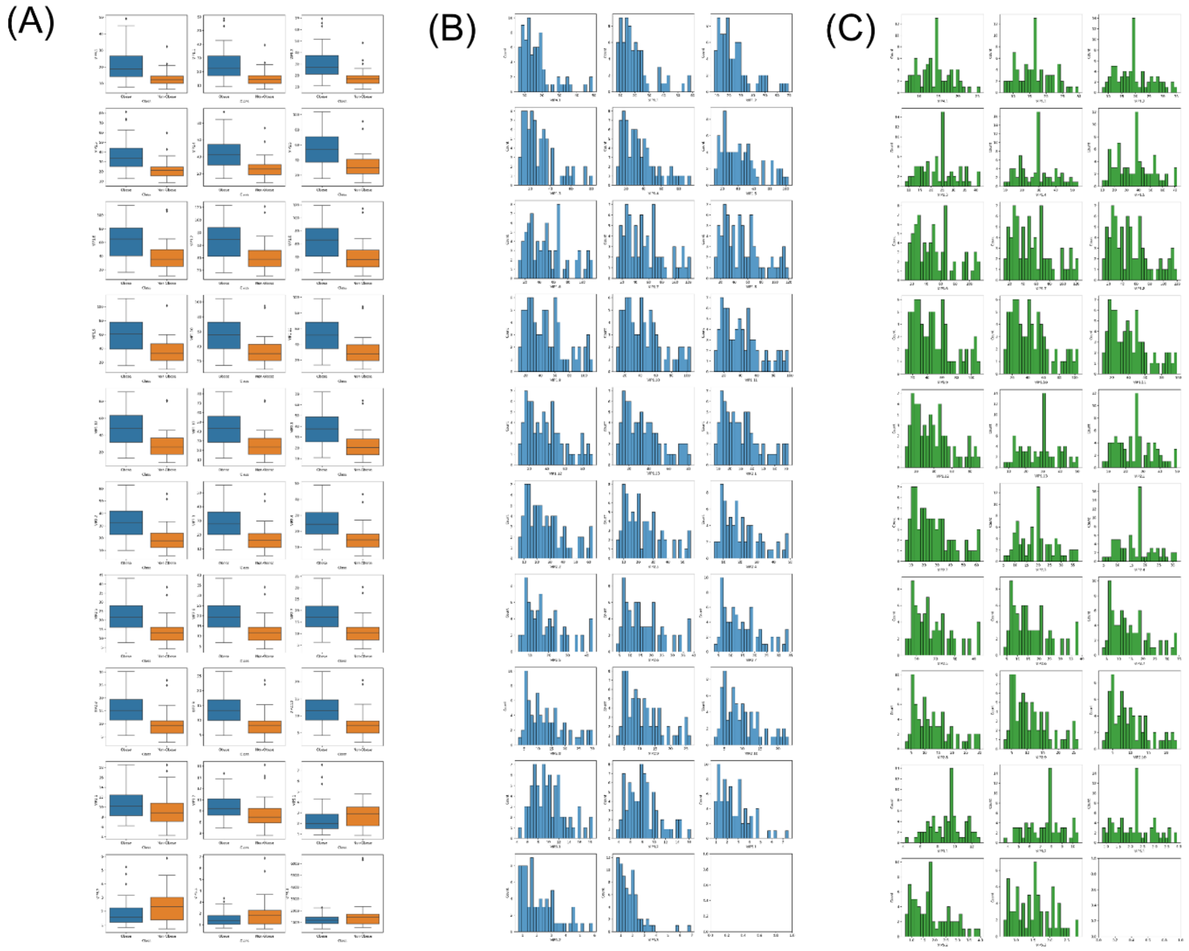

Figure S6: (A) displays the box-plot views of all the NMR signals, highlighting the presence of outliers. (B) showcases the effect of the outliers on the histogram distribution plot of the signals. (C) Illustrates the histogram plot after the outliers have been corrected.

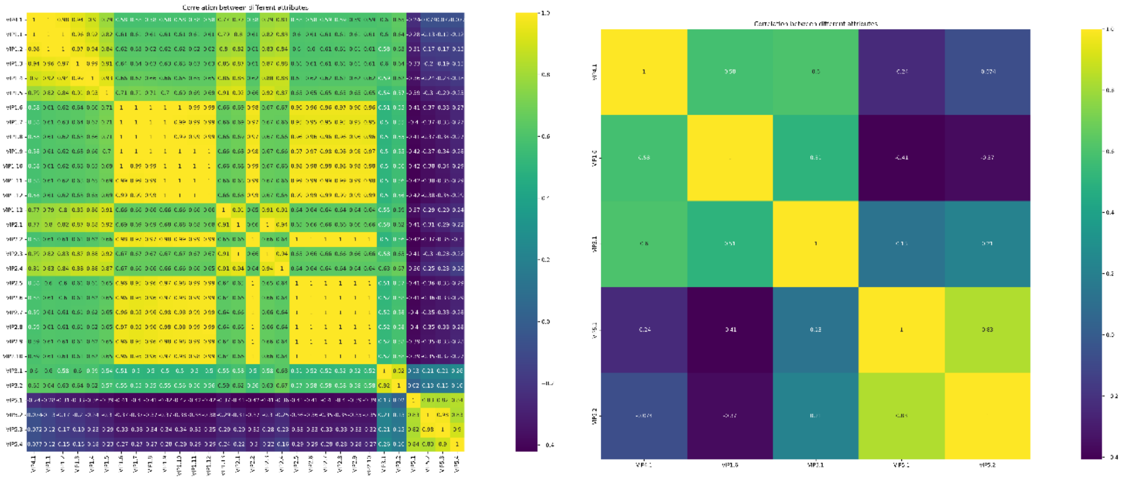

(A) (B)

Figure S7: (A) NMR signals correlation matrix, showing correlation between the signals (B) The reduced set of non-correlated signals for ML study.
